# Targeted Next Generation Sequencing reveals previously unidentified *TSC1* and *TSC2* mutations

**DOI:** 10.1186/s12881-015-0155-4

**Published:** 2015-02-25

**Authors:** Mark Nellist, Rutger WW Brouwer, Christel EM Kockx, Monique van Veghel-Plandsoen, Caroline Withagen-Hermans, Lida Prins-Bakker, Marianne Hoogeveen-Westerveld, Alan Mrsic, Mike MP van den Berg, Anna E Koopmans, Marie-Claire de Wit, Floor E Jansen, Anneke JA Maat-Kievit, Ans van den Ouweland, Dicky Halley, Annelies de Klein, Wilfred FJ van IJcken

**Affiliations:** Department of Clinical Genetics, Ee-2426, Erasmus Medical Center, Wytemaweg 80, 3015 Rotterdam, CN The Netherlands; Center for Biomics, Erasmus Medical Center, Wytemaweg 80, 3015 Rotterdam, CN The Netherlands; Department of Ophthalmology, Erasmus Medical Center, Wytemaweg 80, 3015 Rotterdam, CN The Netherlands; Department of Neurology, Sophia Children’s Hospital, Erasmus Medical Center, Wytemaweg 80, 3015 Rotterdam, CN The Netherlands; Department of Pediatric Neurology, Brain Center Rudolf Magnus, University Medical Center Utrecht, 3508 Utrecht, EA The Netherlands

**Keywords:** Tuberous sclerosis complex, *TSC1*, *TSC2*, HaloPlex, Next Generation Sequencing

## Abstract

**Background:**

Tuberous sclerosis complex (TSC) is an autosomal dominant disorder caused by mutations in *TSC1* and *TSC2*. Conventional DNA diagnostic screens identify a *TSC1* or *TSC2* mutation in 75 - 90% of individuals categorised with definite TSC. The remaining individuals either have a mutation that is undetectable using conventional methods, or possibly a mutation in another as yet unidentified gene.

**Methods:**

Here we apply a targeted Next Generation Sequencing (NGS) approach to screen the complete *TSC1* and *TSC2* genomic loci in 7 individuals fulfilling the clinical diagnostic criteria for definite TSC in whom no *TSC1* or *TSC2* mutations were identified using conventional screening methods.

**Results:**

We identified and confirmed pathogenic mutations in 3 individuals. In the remaining individuals we identified variants of uncertain clinical significance. The identified variants included mosaic changes, changes located deep in intronic sequences and changes affecting promoter regions that would not have been identified using exon-only based analyses.

**Conclusions:**

Targeted NGS of the *TSC1* and *TSC2* loci is a suitable method to increase the yield of mutations identified in the TSC patient population.

**Electronic supplementary material:**

The online version of this article (doi:10.1186/s12881-015-0155-4) contains supplementary material, which is available to authorized users.

## Background

Tuberous sclerosis complex (TSC) is an autosomal dominant disorder characterised by the development of hamartomas in a variety of organs and tissues, including the brain, skin and kidneys [1]. Penetrance is high but the phenotypic manifestations of the disease are variable. Some individuals show only minor signs of disease, sometimes without clear symptoms. Others are severely affected from an early age and at multiple sites throughout the body. Approximately two-thirds of cases are sporadic.

In 75 - 90% of tested individuals categorised as definite TSC according to the 1998 Consensus Conference Clinical Diagnostic Criteria [2], a mutation in either *TSC1* on chromosome 9q34 or *TSC2* on chromosome 16p13.3 is identified [3]. *TSC1* and *TSC2* are tumour suppressor genes that encode respectively hamartin (TSC1; 130 kDa) and tuberin (TSC2; 200 kDa). TSC1 and TSC2 form a stable protein complex that in response to diverse cellular signals, notably growth factors and the availability of energy, regulates the activity of the mechanistic target of rapamycin (mTOR) complex 1 (TORC1) [4]. TORC1 is a central regulator of cell metabolism, controlling protein, lipid and pyrimidine synthesis and autophagy [5]. Elucidation of the role of the TSC1-TSC2 complex in TORC1 signaling has provided new insights into basic cell biology and, importantly for TSC patients, has led to the development of promising new therapies based on the use of specific TORC1 inhibitors such as rapamycin and its derivatives [6].

Early diagnosis of TSC facilitates genetic counselling, therapeutic intervention and disease monitoring [1]. However, the wide variation in the TSC phenotype means that establishing a definite clinical diagnosis of TSC can be challenging, particularly for young patients. The recommendation of the 2013 International TSC Consensus Conference was that the identification of a clearly pathogenic *TSC1* or *TSC2* mutation should be sufficient to make a diagnosis of TSC, even in the absence of clear clinical signs [1]. Unfortunately, despite the remarkable progress in TSC research over the last decade, conventional molecular testing fails to identify a pathogenic *TSC1* or *TSC2* mutation in 10 - 25% of individuals with TSC. These patients are usually referred to as TSC no mutation identified (NMI) [7].

In addition to technical failures, there are several possible reasons for the inability to detect mutations in TSC NMI individuals. First, mutations to other as yet unidentified genes may cause TSC. Second, constitutional epigenetic changes, such as promoter methylation causing transcriptional silencing [8], may occur. Third, specific classes of mutation, such as mosaic mutations and mutations in intronic and regulatory regions may not be detectable using conventional tests. The development of massively parallel sequencing methods, so-called Next Generation Sequencing (NGS) technology, has made it possible to apply new approaches to mutation detection [9], and has the potential to increase the yield of mutations identified in individuals with TSC. High-yield mutation detection methods would help to reduce uncertainty and anxiety in the significant proportion of individuals and families for whom existing diagnostic methods are not informative. NGS strategies have been applied to TSC NMI individuals. For example, in a series of 38 TSC NMI individuals, 2 (6%) mosaic mutations and 5 (13%) heterozygous mutations that had been missed by other mutation detection methods were identified using exon-specific ultra-deep sequencing [10]. One limitation of this approach was that it was restricted to the exons and adjacent intronic sequences of *TSC1* and *TSC2*. Mutations deep within introns or in promoter and other regulatory sequences could not be detected.

Here we present the results of a pilot study using targeted NGS to investigate the DNA of 7 individuals with definite, clinically confirmed TSC in whom extensive conventional mutation analysis had failed to identify a pathogenic mutation. In addition to providing these individuals with increased certainty over their mutation status, exclusion of the existence of pathogenic *TSC1* or *TSC2* mutations in these individuals would help identify a cohort for the identification of the putative *TSC3* locus. We employed the HaloPlex targeted capture method [11]. This technique relies on the specific capture of restriction fragments from the locus of interest followed by amplification and sequencing of the captured fragments. Haloplex has the advantage of working with a defined set of fragments, that simplifies data analysis [12]. In 3 individuals we identified and confirmed a *TSC2* mutation that had been missed, or was undetectable, using conventional exon-based screening approaches. In the remaining individuals we identified novel variants from either the *TSC1* and *TSC2* loci. However, the clinical significance of these variants is not yet certain. Our pilot study indicates that targeted NGS will increase the yield of *TSC1* and *TSC2* mutations identified in the TSC patient population.

## Methods

### Clinical assessment

Clinical information on the 7 index cases and available family members was collected. In each case the proband fulfilled the diagnostic criteria for definite TSC [1] (Table 1). All the individuals included in the study provided informed consent for mutation analysis of *TSC1* and *TSC2*. In each case conventional molecular testing (see below) was negative [13], although multiple neutral *TSC1* and *TSC2* variants were identified in several individuals (Table 2). The study was performed as part of our diagnostic service and was therefore exempt from the Erasmus MC ethics committee approval.

**Table 1:** **Clinical overview of the TSC NMI individuals**

**Individual**	**Age**	**Brain**	**Skin**	**Other**	**Family members**
**I**	**13 yr; Dx0**	**CT**	**HM**	**CR**	Parents (healthy)
**II**	**13 yr; Dx2**	**SEN, CT**	**HM**	**CR, AML**	Parents and sibling (mother and sibling affected)
**III**	**14 yr; Dx4**	**SEN, CT**	**FA, HM**	**AML, RC, CR**	Parents (healthy)
**IV**	**9 yr; ** **Dx8**	**SEGA, SEN, CT**	**FA, HM**	**AML, CR, RP**	Parents (healthy; not investigated clinically)
**V**	**8 yr; ** **Dx3**	**SEN, CT**	**HM**	**AML**	Parents (healthy)
**VI**	**9 yr; ** **Dx1**	**SEN, CT**	**FA, HM**	**AML, CR**	Parents (healthy)
**VII**	**46 yr; Dx15**	**WMA**	**FA, HM**	**TE**	Child (affected)

Age and age at diagnosis (Dx) and the affection status of first degree relatives of the index cases (I - VII) are indicated, as well as the brain, skin and other clinical findings in the index cases. AML: angiomyolipoma; CR: cardiac rhabdomyoma; CT: cortical tuber; FA: facial angiofibroma; HM: hypomelanotic macule; RC: renal cysts; RP: retinal phakoma; SEGA: subependymal giant cell astrocytoma; SEN: subependymal nodule; SP: shagreen patch; TE: tooth enamel defect; UF: ungual fibroma; WMA: white matter abnormalities.

**Table 2:** **Overview of the “new”** ***TSC1*** **and** ***TSC2*** **variants**

**Individual I**	**HaloPlex (% minor allele)**	**Validation (PCR-Sanger sequencing)**			
		**Index**		**Father**	**Mother**
chr9 g.135804394A > G; *TSC1* c.80-55 T > C (intron 3)	+/−	+/−		+/−	−/−
chr9 g.135798153C > T; *TSC1* c.508 + 582G > A (intron 6)	+/−	+/−		−/−	+/−
chr9 g.135791383 T > C; *TSC1* c.738-3539A > G (intron 8)	+/−	+/−		−/−	+/−
chr9 g.135765655 T > C; *TSC1* c.*5967A > G (exon 23; 3′UTR)	+/−	+/−		−/−	+/−
chr 16 g.2108070C > A; *TSC2* c.849-678C > A (intron 9)	+/−	+/−		−/−	+/−
chr 16 g.2119403C > T; *TSC2* c.1717-1054C > T (intron 16)	+/−	+/−		−/−	+/−
chr 16 g.2124981C > G; *TSC2* c.2545 + 591C > G (intron 22)	+/− (20%)	−/−*		−/−	−/−
chr 16 g.2125962C > T; *TSC2* c.2639 + 69C > T (intron 23)	+/−	+/−		−/−	+/−
chr 16 g.2131815insC; *TSC2* c.3814 + 19dup (intron 31)	+/− (18%)	−/−*		−/−	−/−
**Individual II**	**HaloPlex (% minor allele)**	**Validation (PCR-Sanger sequencing)**			
		**Index**	**Sibling**	**Father**	**Mother**
chr9 g.135801283C > T; *TSC1* c.211-157G > A (intron 4)	+/− (31%)	+/−	+/−	+/−	−/−
chr9 g.135775735 T > C; *TSC1* c.2625 + 367A > G (intron 20)	+/−	+/−	+/−	−/−	+/−
chr 9 g.135775718insA (rs36000704); *TSC1* c.2625 + 383dup (intron 20)	+/−	?	?	?	?
chr 9 g.135775530 T > C (rs2284902); *TSC1* c.2625 + 572 (intron 20)	+/−	+/−	+/−	+/−	−/−
chr9 g.135775415dup (rs200047376); *TSC1* c.2625 + 687dup (intron 20)	+/− (35%)	+/−	+/−	+/−	−/−
chr9 g.135775427 T > C (rs6597584); *TSC1* c.2625 + 675 T > G (intron 20)	+/−	+/−	+/−	+/−	−/−
**Individual III**	**HaloPlex (% minor allele)**	**Validation (alle-specific PCR)**			
		**Index**		**Father**	**Mother**
**chr16 g.2129165, rs45464800;** ***TSC2*** **c.3099C > G (exon 16)**	**+/− (9%)**	**+/−**		**−/−**	**−/−**
**Individual IV**	**HaloPlex**	**Validation (PCR-Sanger sequencing)**			
		**Index**		**Father**	**Mother**
**chr16 g.2100489 T > A;** ***TSC2*** **c.225 + 2 T > A (intron 3)**	**+/−**	**+/−**		**−/−**	**−/−**
chr16 g.2102256del10, rs140492671; *TSC2* c.226-1086del10 (intron 3)	+/+	+/+ (ins Alu)		+/− (ins Alu)	+/+ (ins Alu)
**Individual V**	**HaloPlex**	**Validation (PCR-Sanger sequencing)**			
		**Index**		**Father**	**Mother**
chr9 g.135820146delACTCATA; *TSC1* c.-15894_-15888del	+/−	+/−		−/−	+/−
chr9 g.135763459 T > A; 3′ *TSC1* exon 23	+/−	+/−		−/−	+/−
**Individual VI**	**HaloPlex (% minor allele)**	**Validation (alle-specific PCR)**			
		**Index**		**Father**	**Mother**
**chr16 g.2127477G > A;** ***TSC2*** **c.2838-122G > A (intron 25)**	**+/− (12%)**	**+/−**		**−/−**	**−/−**
**Individual VII**	**HaloPlex (% minor allele)**	**Validation (PCR-Sanger sequencing)**			
chr16 g.2101947C > T, rs139385485; *TSC2* c.226-1396 T > C (intron 3)	+/− (18%)	+/−			
chr16 g.2102256del10, rs140492671; *TSC2* c.226-1086del10 (intron 3)	+/− (10%)	+/− (ins Alu)			
chr16 g.2105289 T > C, rs77037371; *TSC2* c.482-114 T > C (intron 5)	+/− (70%)	+/−			
chr16 g.2105335C > G, rs2516734; *TSC2* c.482-68C > G (intron 5)	+/− (70%)	+/−			
chr16 g.2113125A > G; *TSC2* c.1443 + 71A > G (intron 14)	+/−	+/−			
chr16 g.2113464C > T; *TSC2* c.1443 + 410C > T (intron 14)	+/−	+/−			
chr16 g.2120785C > T; *TSC2* c.1839 + 206C > T (intron 17)	+/−	+/−			
chr16 g.2130697G > A; *TSC2* c.3610 + 319G > A (intron 30)	+/−	+/−			

Variants identified by HaloPlex custom capture NGS without an rs-number in dbSNP132 are listed per individual. The results of the subsequent PCR-Sanger sequencing and/or allele-specific PCR validation are shown for the relevant individual and any available family members. Results for variants identified with a skewed allelic ratio (minor allele detected in <40% or >60% of the sequence reads) are also shown. * indicates a discrepancy between the HaloPlex result and the validation experiment. ? indicates that the nucleotide change identified by HaloPlex could not be confirmed by Sanger sequencing due to the presence of polyA:T stretches. Pathogenic mutations are indicated in bold.

### DNA and RNA isolation

DNA extraction from peripheral blood was performed according to standard protocols. DNA quality and concentration were checked with the Quant-iT PicoGreen dsDNA Kit (Invitrogen Corporation, Carlsbad, CA, USA). DNA and RNA isolation from skin fibroblasts in culture was performed according to standard protocols. Cells were cultured under standard conditions, and in the presence of cycloheximide, to inhibit nonsense-mediated mRNA decay.

### Conventional *TSC1* and *TSC2* molecular testing

Conventional mutation analysis of *TSC1* and *TSC2* was performed as described previously [12,13]. For the detection of single nucleotide changes, denaturing gel electrophoresis (DGGE), single-strand conformation polymorphism (SSCP) and/or direct sequence analysis of all coding exons and exon/intron boundaries was performed. For the detection of large rearrangements, Southern blotting, fluorescence *in situ* hybridisation (FISH), quantitative (Q)-PCR and/or multiplex ligation-dependent probe amplification (MLPA) were performed.

### HaloPlex design

We designed a HaloPlex custom capture array using the SureDesign software provided by Agilent Technologies (Santa Clara, USA). The design was tailored to the 150 base-pair (bp) paired-end sequencing technology from Illumina (San Diego, USA) and targeted 64626 bases encompassing the *TSC1* locus (GRCh37/hg19 chromosome 9q34: g.135825221 - 135760595; bases covered: 63855 bp (98.8%); bases not covered: 741 bp) and 52228 bases encompassing the *TSC2* locus (GRCh37/hg19 chromosome 16p13.3: g.2087907 - 2140135; bases covered: 50997 bp (97.6%); bases not covered: 1231 bp). The *TSC1* and *TSC2* loci were digested into respectively 1338 and 1074 restriction fragments for capture and amplification (size range 50–450 bp). Amplicons could not be designed for 741 bases (1.1%) of the *TSC1* locus and for 1231 bases (2.4%) of the *TSC2* locus (Additional file 1: Table S1). To allow identification of mutations affecting promoters and other 5′ regulatory elements, ~10 kilobases (kb) upstream of *TSC1* and *TSC2* were captured. In addition, ~10 kb downstream of the *TSC1* 3′-UTR was captured to allow the detection of mutations affecting downstream regulatory sequences. For the *TSC2* locus, downstream sequences were not included. This region, encompassing the 3′ end of the adjacent *PKD1* gene, includes sequences that are repeated at high homology elsewhere on chromosome 16 [14] and were considered likely to complicate target capture and data analysis. Furthermore, mutations affecting *PKD1* as well as *TSC2* usually result in a distinct, severe renal phenotype [15]. None of the patients included in our study had severe renal involvement from an early age (Table 1).

### Sequence data generation and analysis

Sequence data was generated with an Illumina MiSeq sequencer, using the paired-end 150 bp sequencing protocol and the MiSeq reagent kit V2. Prior to alignment, the TruSeq 3′ adapter sequences were trimmed from the reads using custom in-house software that matches the largest possible sub-sequence of the adapter to that of the read and then trims the matching and downstream sequence. The trimmed reads were subsequently aligned to the human reference genome (build hg19) using the Burrows-Wheeler alignment (BWA) tool [16] and custom-designed in-house alignment software called NIMBUS (Brouwer *et al*., in preparation). Single nucleotide substitution (SNP) and insertion/deletion (InDel) variants were called using two strategies. To identify germ-line variants, the Genome Analysis Tool Kit (GATK) and Unified Genotyper [17] were used. Variants were assessed according to the depth of coverage (number of reads and number of HaloPlex fragments containing the variant base), the number of individuals with the same variant, whether the variant corresponded to a known SNP and whether the variant was likely to affect either the coding sequence or expression of *TSC1* or *TSC2*. To call mosaic variants, SAMtools mpileup [18] and in-house software were used. We considered all variants called at a minor allele frequency >10% and with at least 3 reads representing an alternative allele. To compare the coverage per target fragment per individual we calculated z-scores for all the sequenced fragments in the target regions [19].

To investigate potential effects on splicing, the identified variants were analysed with SpliceSiteFinder-like, MaxEntScan, NNSPLICE, GeneSplicer and Human Splice Finder in the ALAMUT version 2.3 software package (Interactive Biosoftware, Rouen, France).

### PCR-based confirmation of identified variants

Allele-specific PCR was performed according to a standard protocol (conditions and primer sequences available on request). RT-PCR was performed on total RNA isolated from untreated and cycloheximide-treated cultures of human skin fibroblasts. Standard PCR and Sanger sequencing were performed as described previously [12] (primer sequences available on request).

## Results and discussion

### HaloPlex custom capture of the *TSC1* and *TSC2* genomic loci

**Figure 1:**
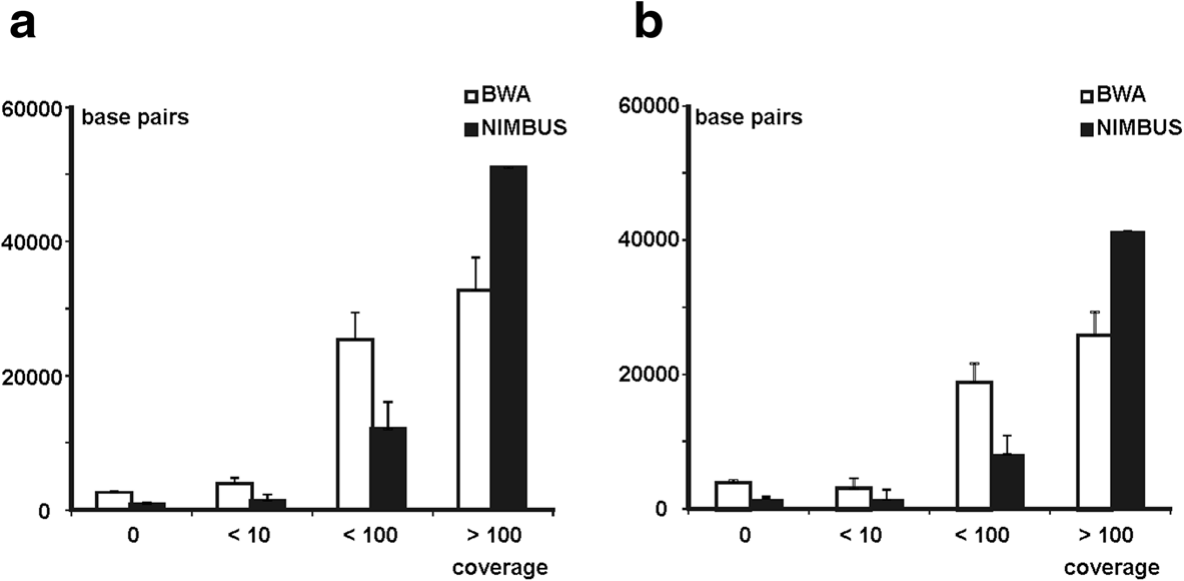
**Coverage expressed as number of reads (forward + reverse) per base.** Bases with >100 reads were considered to be adequately covered; coverage was inadequate for bases where the number of reads was <10. **a**. *TSC1;* **b**. *TSC2.*

The *TSC1* and *TSC2* loci of blood DNA samples from 7 clinically definite TSC patients were analysed. We mapped the sequence reads using two different alignment tools: a standard BWA analysis [16] and NIMBUS, an in-house custom-designed aligner (Brouwer *et al*., in preparation). A comparison of these two approaches is shown in Figure 1. Using standard BWA we obtained an average of 292487 reads per individual; using NIMBUS we obtained an average of 297476 reads per individual (Additional file 1: Table S2). For both aligners, the mean coverage for the *TSC1* and *TSC2* loci was 96% and 92% respectively at a read-depth >0. At a read-depth >10, the mean coverage fell to 90% for *TSC1* and 86% for *TSC2* using BWA, and 97% for *TSC1* and 94% for *TSC2* using NIMBUS (Additional file 1: Table S2). At a read-depth >100, mean coverage fell to 50% for both loci using BWA, while with NIMBUS, on average, 79% of both loci was covered (Figure 1). Detection of allelic imbalances and mosaic mutations was relatively straight-forward for these regions. All variants (see below, and Additional file 1: Table S3), were detected by both approaches. Approximately 10% of the *TSC1* locus and 14% of the *TSC2* locus were covered by <10 reads (Additional file 1: Table S1), and included part of *TSC2* exon 34 (chr16: 2134380–2134393) and part of *TSC1* exon 10 (chr9: 135786880–135787089). For these regions it is possible that germ-line mutations were missed.

To try and identify regions showing possible copy number variations we calculated z-scores for all the sequenced fragments in the target regions, but did not find evidence for large (>150 basepair) deletions or other rearrangements in the 7 TSC NMI individuals.

### *TSC1* locus

We identified between 0 and 74 heterozygous SNPs, as listed in dbSNP132, across the *TSC1* locus per individual (mean >33 SNPs per individual), and between 3 and 25 SNPs homozygous for the minor allele (mean >15 per individual) (Additional file 1: Table S3A). Furthermore, we identified between 1 and 7 InDel variants (mean 3 per individual), as listed in dbSNP132, and identified 4 variants that were not listed in dbSNP132 but had been reported in the GoNL database [20]. In addition to the variants listed in dbSNP132 and the GoNL database, we identified 6 variants that had been identified previously in 3 of the 7 individuals by conventional molecular testing, but had been excluded as being disease-causing. All of the *TSC1* variants identified by previous molecular testing were confirmed by the HaloPlex data. In 4 individuals, previous molecular tests had not identified any *TSC1* variants. Finally, we identified 10 variants that had not, to our knowledge, been reported previously. We considered these “new” variants as candidate TSC-causing mutations (see below).

### *TSC2* locus

Across the *TSC2* locus we identified between 2 and 40 heterozygous SNPs per individual(mean >18 SNPs per individual), between 0 and 19 SNPs homozygous for the minor allele (mean >4 per individual), and between 0 and 5 InDel variants (mean >2 per individual) (Additional file 1: Table S3B). In addition, 2 variants listed in the GoNL database were identified. Previous molecular testing had identified 21 SNPs and 10 InDels in 4 of the individuals tested. All these variants had been excluded as being disease-causing. All of the *TSC2* variants identified by previous molecular testing were confirmed by the HaloPlex data. In 3 individuals, previous molecular tests had not identified any *TSC2* variants. We identified 13 “new” *TSC2* variants that had not, to our knowledge, been reported previously and that we considered as potential TSC-causing mutations (see below).

### Individual I

Individual I was diagnosed with definite TSC on the basis of cardiac rhabdomyoma, hypomelanotic macule and cortical tuber (Table 1). Clinical examination of the parents did not reveal signs of TSC. We identified 4 “new” variants from the *TSC1* locus and 5 “new” variants from the *TSC2* locus in DNA from individual I (Table 2). We could validate all the “new” *TSC1* and 3/5 “new” *TSC2* variants by Sanger sequencing. In each case the variant was also detected in DNA from one of the individual’s parents (Table 2). One variant, *TSC1* c.738-3539A>G (intron 8; chr9 g.135791383T>C), was predicted to create a new splice acceptor site. However, we were unable to detect any abnormal *TSC1* splice products by RT-PCR analysis of RNA isolated from cultured skin fibroblasts of individual I (data not shown). No effects on splicing were predicted for the other “new” variants.We could not confirm the presence of 2 *TSC2* variants, either by PCR followed by Sanger sequencing, or by allele-specific PCR (Table 2). The *TSC2* c.2545+591C>G (intron 22; chr16 g.2124981C>G) change was identified in 2/10 reads using the BWA tool, and in 4/12 reads using NIMBUS. The same variant was identified at a similar frequency in the other 6 individuals tested, and maps to a region consisting of seven 29 base pair repeats, making HaloPlex capture as well as alignment and variant calling challenging. The *TSC2* c.3814+19dup (intron 31; chr16 g.2131816insC) change was identified in 54/305 reads (18%) using BWA and 18/437 reads (4%) with NIMBUS. For the other individuals tested, between 0% and 6% of the reads at this position had the same insertion, possibly due to stuttering of the polymerase over the adjacent poly G stretch. We concluded that the *TSC2* c.2545+591C>G and *TSC2* c.3814+19dup variants were most likely artefacts of the NGS procedure, and unlikely to be the causative mutation in individual I. No effects on splicing were predicted for either variant. We consider it unlikely that any of the other variants identified in DNA from individual I (Table 2) are pathogenic mutations.

### Individual II

Individual II was diagnosed with definite TSC on the basis of cardiac rhabdomyoma, hypomelanotic macules, angiomyolipoma, subependymal nodules and cortical tubers (Table 1). No clinical data was available for the individual’s parents, but the mother was reported to have TSC. One sibling had died at birth due to the presence of cardiac rhabdomyoma, and hypomelanotic macules and cortical tubers were reported in another, younger sibling. We identified 5 variants mapping to *TSC1* intron 20 in individual II: a “new” variant, *TSC1* c.2625+367A>G and 4 SNPs, rs36000704 (*TSC1* c.2625+383dup), rs2284902 (*TSC1* c.2625+572A>G), rs200047376 (*TSC1* c.2625+687dup) and rs6597584 (*TSC1* c.2625+675T>G)(Table 2). We performed PCR followed by Sanger sequencing to confirm the presence of these variants in DNA from individual II, individual II’s younger sibling and their parents. We could not confirm the *TSC1* c.2625+383dup SNP (rs36000704) because we were unable to obtain satisfactory Sanger sequence data due to the presence of an extensive polyA:T tract. SNPs rs2284902, rs6597584, rs200047376 and rs150221955 were present in the father and both children, while the *TSC1* c.2625+367A>G variant was present in DNA from the mother and both children. This variant was predicted to create a new splice donor site. However, we did not have access to any RNA samples from this family. Therefore, we classified the *TSC1* c.2625+367A>G change as a variant of uncertain clinical significance.

### Individual III

**Figure 2:**
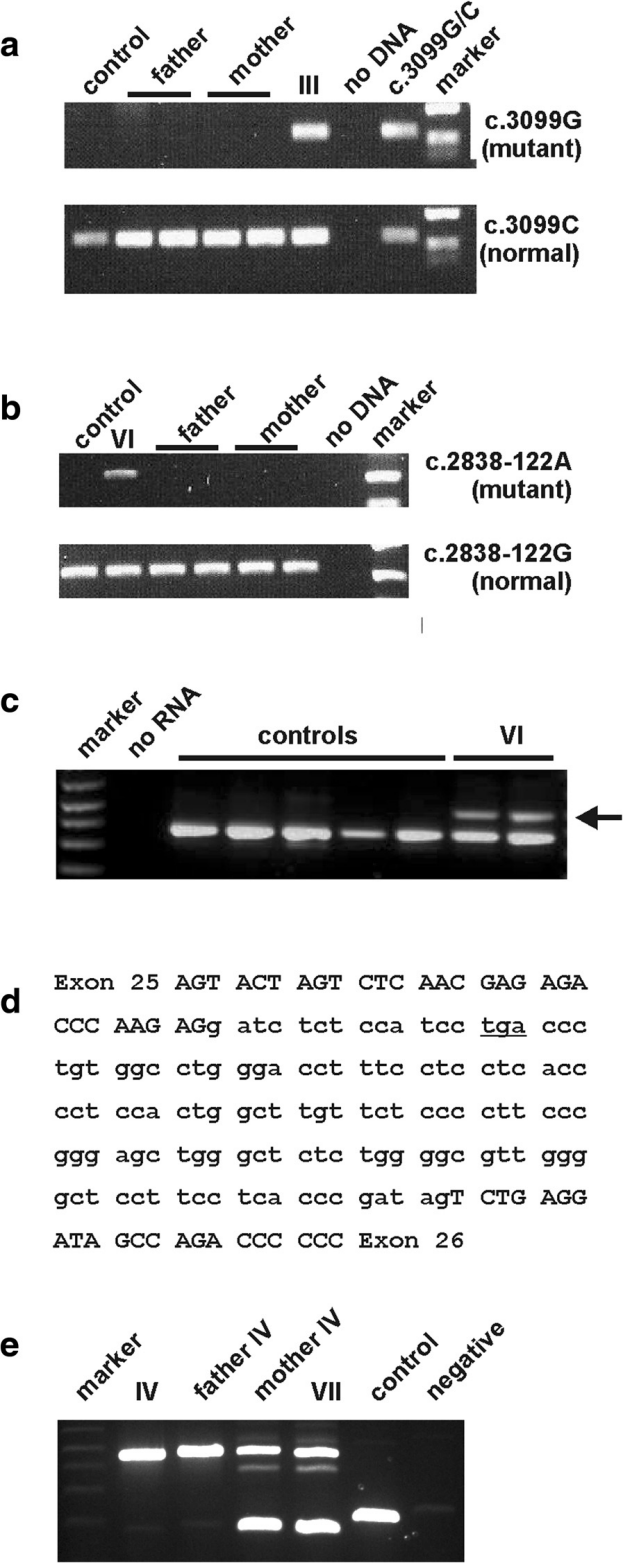
**Allele-specific amplification of** ***TSC2*** **mosaic variants. a**. Allele-specific amplification of the *TSC2* c.3099C>G (p.Y1033*) mosaic variant**.** Specific amplification of the mutant c.3099G (upper panel) and normal c.3099C (lower panel) alleles from DNA from an unrelated healthy individual (control), DNA from individual III (III), both parents, and an individual with TSC heterozygous for the *TSC2* c.3099C>G (p.Y1033*) pathogenic variant (c.3099C/G). **b**. Allele-specific amplification of the *TSC2* c.2838-122G>A mosaic variant. Specific amplification of the mutant **c**.2838-122A (upper panel) and normal c.2838-122G (lower panel) alleles from DNA from an unrelated healthy individual (control), individual VI (VI) and both parents. **c**. RT-PCR of *TSC2* mRNA from individual VI (VI) and 5 unrelated individuals (controls). An extra splice variant was amplified from RNA from individual VI (arrow), but not from the controls. **d**. Sequence of the additional RT-PCR product identified in individual VI (see Figure 2c, arrow). Sequences derived from exons 25 and 26 are in capitals; the premature stop codon is underlined. **e**. PCR amplification of the *TSC2* c.226-1086del10 (intron 3; chr16 g.2102256del10) variant (rs140492671) from DNA from individuals IV and VII, the parents of individual IV and DNA from an unrelated healthy individual (control).

Individual III was diagnosed with TSC on the basis of angiomyolipoma, renal cysts, cardiac rhabdomyoma, facial angiofibroma, hypomelanotic macule, subependymal nodules and cortical tubers (Table 1). No signs of TSC were reported in the parents. The SAMtools-based variant caller identified SNP rs45464800 (*TSC2* exon 16 c.3099, chr16 g.2129165) as heterozygote in individual III. Closer inspection of the sequence data at this position revealed 984 (BWA) or 1078 (NIMBUS) reads with a C and 103 (BWA) or 110 (NIMBUS) reads with a G. The *TSC2* c.3099C>G change creates a new stop codon, resulting in premature termination of the *TSC2* open reading frame (p.Y1033*). To confirm the presence of the *TSC2* c.3099C>G change in DNA from individual III, we performed standard PCR followed by Sanger sequencing as well as allele-specific PCR amplification. Sanger sequencing was inconclusive. Although differences in the C:G peak ratios were detected in the sequence traces from individual III compared to either parent, they were not considered sufficient to confirm the presence of the variant in individual III (Additional file 1: Table S4). In contrast, allele-specific PCR clearly revealed the presence of the mutant allele in DNA from individual III, but not in DNA from either parent (Figure 2a). We concluded that the mosaic *TSC2* c.3099C>G (p.Y1033*) change was the pathogenic mutation in individual III.

### Individual IV

Individual IV was diagnosed with TSC due to angiomyolipoma, cardiac rhabdomyoma, retinal phakoma, facial angiofibroma, hypomelanotic macules, subependymal nodules, subependymal giant cell astrocytoma and cortical tubers (Table 1). Clinical examination of the parents did not reveal signs of TSC. We identified one “new” heterozygous *TSC1* variant and 6 “new” heterozygous variants in *TSC2*. One of these, *TSC2* c.225+2T>A (chr16 g.2100489T>A) was predicted to destroy the splice donor site at the 3′ end of exon 3. This change was not detected in DNA from individual IV’s parents and we concluded that this was the pathogenic mutation in individual IV.

### Individual V

Individual V was diagnosed with TSC on the basis of hypomelanotic macules, subependymal nodules, cortical tubers and a possible renal angiomyolipoma (Table 1). No signs of TSC were found in the parents, but epilepsy was reported in a paternal cousin of the mother. We identified 2 “new” variants at the *TSC1* locus: a 7 bp deletion 206 bp upstream of *TSC1* exon 1 (*TSC1* c. -15894_-15888delTATGAGT; chr9 g.135820146delACTCATA) and a chr.9 g.135763459T>A substitution downstream of *TSC1* exon 23 (Table 2). The presence of both variants in DNA from individual V and individual V’s mother was confirmed by Sanger sequencing. Neither variant was detected in DNA from individual V’s father. The *TSC1* c. -15894_-15888delTATGAGT deletion destroys a putative MYB transcription factor binding site within the region of maximal *TSC1* promoter activity [21]. Previously, we identified a *TSC1* c.-16116_-15364del753 mutation in an individual with TSC [13]. Although both deletions affect the same core promoter region, individual V’s mother, who carries the *TSC1* c.-15894_-15888delTATGAGT variant, had no signs of TSC. No RNA or additional DNA samples were available from this family and in the absence of any other findings, we consider the *TSC1* c.-15894_-15888delTATGAGT deletion to be a variant of uncertain clinical significance.

### Individual VI

Individual VI was diagnosed with TSC on the basis of bilateral, multiple renal angiomyolipoma, facial angiofibroma, hypomelanotic lesions, cardiac rhabdomyoma, subependymal nodules and subcortical tubers. Clinical examination of the parents did not reveal signs of TSC. A *TSC2* c.2838-122G>A (chr16 g.2127477G>A) change was identified in ~12% of the sequence calls at this position (BWA: 21 reads A, 147 reads G; NIMBUS: 21 reads A, 151 reads G) in DNA from individual VI. The *TSC2* c.2838-122G>A change was predicted to create a new splice acceptor site 120 bases upstream of the alternatively spliced *TSC2* exon 26 [22]. To confirm the presence of the *TSC2* c.2838-122G>A change in DNA from individual VI, we performed Sanger sequencing as well as allele-specific PCR. Sanger sequencing was inconclusive. Although differences in the G:A peak ratios were detected in the sequence traces from individual VI compared to either parent, they were not considered sufficient to confirm the presence of the variant in individual VI (Additional file 1: Table S4). In contrast, allele-specific PCR clearly revealed the presence of the variant in DNA from individual VI, but not in DNA from either parent (Figure 2b). To investigate whether the identified change affected splicing, RT-PCR was performed on RNA isolated from skin fibroblasts from individual VI. Compared to control samples, an additional PCR product was amplified from RNA from individual VI (Figure 2c). Sequence analysis revealed that this product contained an insertion of the 120 nucleotides immediately upstream of exon 26, resulting in premature truncation of the *TSC2* open reading frame (Figure 2d). We concluded that the mosaic *TSC2* c. 2838-122G>A change was most likely responsible for TSC in individual VI.

### Individual VII

Individual VII was diagnosed with TSC on the basis of hypomelanotic macules, facial angiofibroma, tooth enamel defects, brain white matter abnormalities detected by radiology and an affected child with subependymal nodules, cortical tuber, cardiac rhabdomyoma and hypomelanotic macules (Table 1). We identified 4 “new” variants in *TSC2*, in introns 14 (c.1443+71A>G, chr16 g.2113125A>G; c.1443+410C>T, chr16 g.2113464C>T), 17 (c.1839+206C>T, chr16 g.2120785C>T) and 30 (c.3610+319G>A, chr16 g.2130697G>A). In addition, 4 variants (in introns 3 and 5) were identified where the allelic ratio was skewed (minor allele detected in <40% or >60% of the sequence reads) (Table 2). For 3 of these, the presence of both alleles in DNA from individual VII was confirmed by PCR followed by Sanger sequencing. The remaining variant (rs140492671; *TSC2* c.226-1086delTTGTCTGAAT (intron 3), chr16 g.2102256-2102266delTTGTCTGAAT) was identified in 10% of the reads. Individual IV (see above) was homozygous for rs140492671. To confirm the presence of rs140492671 in DNA from individuals IV and VII, we performed PCR followed by Sanger sequencing. In individual VII, PCR revealed a larger than expected ~450 bp product in addition to the expected ~190 bp product. DNA from individual IV and individual IV’s father showed only the ~450 bp product, while PCR of DNA from individual IV’s mother revealed both bands (Figure 2e). Sequence analysis of the ~190 bp product from individual IV’s mother and control DNA samples, revealed the normal sequence. Nucleotides 2102256–2102266 were not deleted. Sequence analysis of the ~450 bp band from individuals IV, VII and individual IV’s parents revealed the presence of a 338 bp insertion consisting of an Alu repeat flanked by two direct 18 bp repeat sequences (5′-AAGAGTATTGTCAATGAG-3′). A *de novo TSC2* c.225+2T>A mutation was identified in individual IV (see above); therefore the presence of the Alu insertion in individual IV and both unaffected parents indicates that it is unlikely to cause TSC in indiviual VII. In conclusion, we identified 4 variants of uncertain clinical significance in DNA from individual VII, but failed to identify a good candidate *TSC1* or *TSC2* mutation. It is possible that we missed a mosaic mutation in individual VII. A DNA sample from the affected child of individual VII has been requested. Analysis of this sample might help identify the causative mutation in this family.

## Conclusions

Identification of a *TSC1* or *TSC2* mutation is sufficient for a diagnosis of TSC and provides individuals and families affected by TSC with clarity regarding their risk of developing symptoms, or of having an affected child. To increase the yield of *TSC1* and *TSC2* mutations identified in the TSC patient population we applied a targeted NGS strategy to assess the *TSC1* and *TSC2* loci in 7 TSC NMI individuals. In total, we identified and confirmed 19 “new” variants (Table 2). These have all been submitted to the *TSC1* and *TSC2* Leiden Open Variation Databases (http://chromium.liacs.nl/LOVD2/TSC/home.php). Sequence analysis of the *TSC1* and *TSC2* genomic loci in a larger cohort of individuals with TSC could help establish whether any of these changes are disease-causing or are more likely to be rare, benign variants. In addition, functional studies could help determine whether the variants affect *TSC1* or *TSC2* expression. The availability of patient-derived cells for RNA-based studies would greatly facilitate these studies. In addition, DNA from both biological parents and other family members (affected and unaffected) will be helpful for establishing pathogenicity. We identified “new” variants in all individuals tested, and it is likely that many other individuals will carry other new variants of uncertain clinical significance. In the absence of an obvious candidate pathogenic mutation, it will be even more important to be able to perform functional studies that can distinguish pathogenic and benign variants.

We failed to validate 4 variants. In 2 cases (*TSC2* c.2545+591C>G and c.3814+19dup; individual I) we concluded that these were miscalls in regions with low coverage; in another case (rs36000704; individual II), the presence of a polyA:T tract made confirmation with Sanger sequencing impossible; and in the final case (rs140492671; individuals IV and VII), there was a discrepancy between the HaloPlex data and the PCR and Sanger sequencing results.

For the *TSC1* locus we obtained >90% coverage at a read-depth >10 (Additional file 1: Tables S1 and S2) and identified 21–91 variants per individual (Additional file 1: Table S3A). In individual I and the unaffected mother, we identified a substitution in intron 8 (*TSC1* c.738-3539A>G) that was predicted to create a new 3′ splice site. However, we did not identify abnormal splice products in RNA from individual I. In individual II and the affected sibling and mother, we identified a novel *TSC1* c.2625+367A>G variant in intron 20 that was predicted to create a new 5′ splice site. Unfortunately, we were unable to investigate whether this variant affects splicing of the *TSC1* mRNA and the clinical significance of the variant remains uncertain, even though it cosegregated with TSC. In individual V and the unaffected mother, we identified a 7 bp deletion in the *TSC1* promoter (*TSC1* -15894_-15888delTATGAGT) that affects a predicted binding site for c-MYB [21] (http://alggen.lsi.upc.es/). c-MYB has been found to interact with the *TSC1* promoter, as well as the *TSC2* and *TBC1D7* promoters [23], and although it is predominantly expressed in hematopoietic cells, other tissues, such as skin, are known to express c-MYB (http://www.ebi.ac.uk/gxa/genes/ENSG00000118513). It will be important to establish whether the 7 bp deletion affects *TSC1* expression. However, in the absence of additional clinical or functional findings, we have not yet been able to classify this variant.

Over 86% of the *TSC2* locus was covered by 10 or more reads (Additional file 1: Tables S1 and S2) and 10–46 variants were identified per individual (Additional file 1: Table S3B). In individual IV we identified a *de novo* splice site mutation in intron 2 (*TSC2* c.225+2T>A) that had been missed during the original screening procedure. Other examples of TSC NMI individuals with germline, exonic mutations that have been missed by conventional molecular screening have been described previously [10]. In 2 individuals we detected mosaic *TSC2* mutations. In individual III a *TSC2* c.3099C>G (p.Y1033*) mutation was identified in 10% of the corresponding sequence reads, and in individual VI a novel *TSC2* c.2838-122G>A variant was identified in 12% of the corresponding sequence reads. The *TSC2* c.2838-122G>A variant was predicted to create a new splice acceptor site, 120 nucleotides upstream of the normal exon 26 acceptor site. RT-PCR analysis of RNA isolated from skin fibroblasts of individual VI revealed the presence of an additional splice product (Figure 2c) and sequence analysis confirmed the insertion of 120 nucleotides, resulting in a premature stop codon (Figure 2d). The presence of both variants was confirmed by allele-specific PCR (Figures 2a and b), but neither variant could be reliably detected with a standard PCR-Sanger sequencing approach (Additional file 1: Table S4). We selected TSC NMI individuals with clinically definite TSC. Therefore our cohort is not representative of the TSC NMI population as a whole [7,12]. Nonetheless, we note that both individuals with a mosaic *TSC2* mutation had TSC with multiple organ involvement, but mild or no intellectual disability.

We did not obtain equal or optimal coverage of the entire *TSC1* and *TSC2* genomic regions. Coverage varied from 0 to >1000 calls per nucleotide. It is possible that in regions with <100 calls per nucleotide we missed mosaic mutations and that in low coverage regions (<10 calls per nucleotide) we also missed germ-line mutations. We are now working on improving our design, to utilise longer reads and alternative HaloPlex probe sets to obtain higher overall coverage and, specifically, higher coverage of the coding sequences. In addition, analysis of patient DNA from other easily accessible tissue sources, such as buccal or skin cells, might be useful to help identify mutations that are only present in a subset of somatic cells.

**Figure 3:**
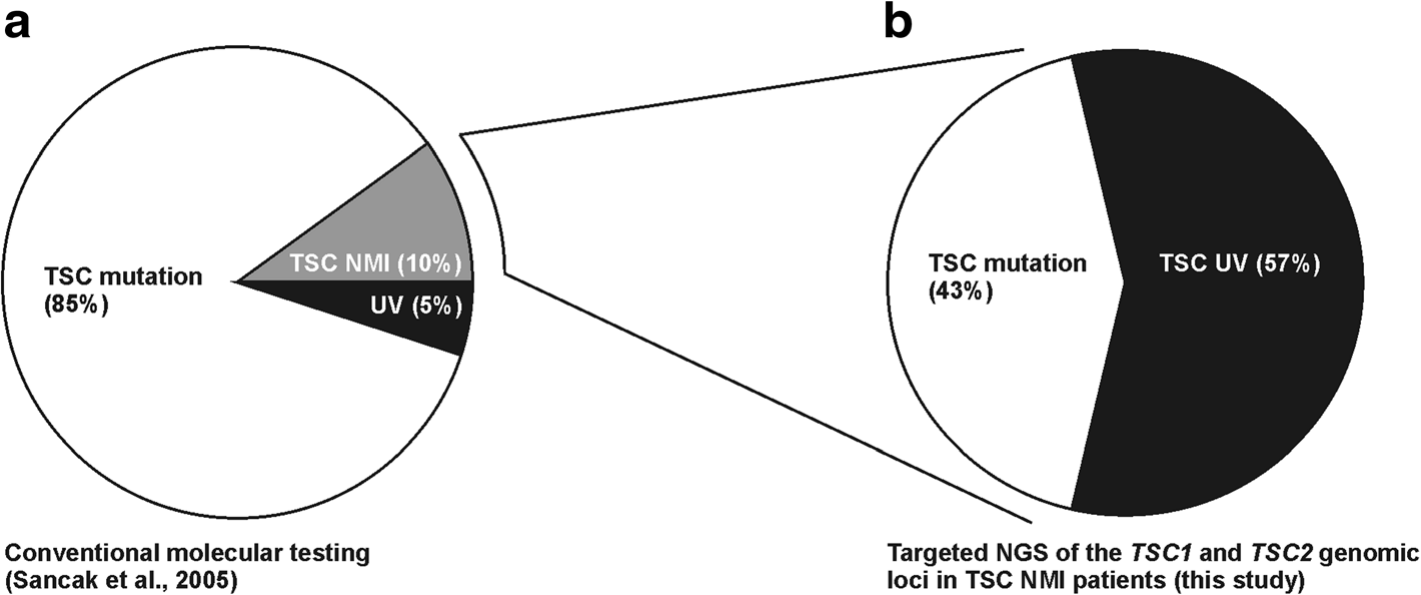
**Pie charts showing the diagnostic yield in individuals with TSC.** Percentages of individuals with definite TSC and a pathogenic *TSC1* or *TSC2* mutation (TSC mutation), an unclassified variant (UV) or no mutation identified (NMI) are indicated. **a**. Results of conventional molecular testing in individuals with definite TSC [12]. **b**. Results of targeted NGS of the *TSC1* and *TSC2* loci in individuals classified as TSC NMI after conventional molecular testing (this study).

In summary, we screened 7 TSC NMI individuals using a HaloPlex custom capture NGS approach. In individual IV we identified a germ-line mutation that had been missed by previous mutation analysis screens; in individuals III and VI we identified and verified mosaic mutations; and in individuals I, II, V and VI we identified variants of uncertain clinical significance. In individual VI the mutation was located outside the region screened in our standard protocol. In total, we confirmed pathogenic mutations in 3/7 (43%) TSC NMI individuals (Figure 3), indicating that targeted NGS of the *TSC1* and *TSC2* loci is a useful technique for the identification of otherwise difficult to detect *TSC1* and *TSC2* mutations in individuals with TSC. The challenge will be to ensure that targeted NGS-based detection is also a cost-effective alternative for mutation screening in TSC. Currently we have a cohort of >40 TSC NMI individuals in whom we would like to perform targeted NGS of the *TSC1* and *TSC2* loci. We predict that the implementation of this technique in routine DNA diagnostic screens will result in an increased yield of *TSC1* and *TSC2* mutations in individuals with TSC.

## Additional file

Additional file 1: Table S1. Overview of low coverage regions for Haloplex targeted genomic sequencing of the *TSC1* and *TSC2* loci. **Table S2.** Haloplex custom capture targeted sequencing reads mapped to the *TSC1* and *TSC2* loci using NIMBUS and standard BWA alignment. **Table S3.** Overview of Haloplex targeted genomic sequencing of the *TSC1* and *TSC2* loci. **Table S4.** Peak ratios Sanger sequencing.
