# Supplementary material for: Targeted Next Generation Sequencing reveals previously unidentified TSC1 and TSC2 mutations
Source: BMC Med Genet. 2015 Feb 25;16:10. doi: 10.1186/s12881-015-0155-4 (PMC4422413; doi:10.1186/s12881-015-0155-4)
Supplement: Additional file 1: Table S1. — Overview of low coverage regions for Haloplex targeted genomic sequencing of the TSC1 and TSC2 loci. Table S2. Haloplex custom capture targeted sequencing reads mapped to the TSC1 and TSC2 loci using NIMBUS and standard BWA alignment. Table S3. Overview of Haloplex targeted genomic sequencing of the TSC1 and TSC2 loci. Table S4. Peak ratios Sanger sequencing. [file 12881_2015_155_MOESM1_ESM.docx]

**Supporting Material**

**Supporting Table S1**: **Overview of low coverage regions for Haloplex targeted genomic sequencing of the *TSC1* and *TSC2* loci.** Regions of the *TSC1* and *TSC2* loci predicted not to be covered by the Haloplex targeted genomic design (S1A: Expected low coverage), and the maximum extents of the regions from the *TSC1* and *TSC2* loci showing <10 x coverage after Haloplex targeted genomic sequencing (S1B: Observed low coverage) are listed. Nucleotide numbering is according to human genome reference build GRCh37/hg19; start and end positions and size in basepairs (bp) of the low coverage regions are indicated.

**S1A: Expected low coverage.**

| ***TSC1*** | | | ***TSC2*** | | |
| --- | --- | --- | --- | --- | --- |
| **start** | **end** | **size (bp)** | **start** | **end** | **size (bp)** |
| 135760748 | 135760757 | 9 | 2091682 | 2091691 | 9 |
| 135764927 | 135764928 | 1 | 2091890 | 2091905 | 15 |
| 135765509 | 135765513 | 4 | 2097228 | 2097235 | 7 |
| 135766091 | 135766100 | 9 | 2099291 | 2099322 | 31 |
| 135785570 | 135785579 | 9 | 2101075 | 2101140 | 65 |
| 135789256 | 135789368 | 112 | 2105658 | 2105681 | 23 |
| 135790018 | 135790237 | 219 | 2107778 | 2107781 | 3 |
| 135802111 | 135802229 | 118 | 2109262 | 2109499 | 237 |
| 135805680 | 135805764 | 84 | 2109804 | 2109815 | 11 |
| 135808539 | 135808639 | 100 | 2114968 | 2115183 | 215 |
| 135812528 | 135812547 | 19 | 2115334 | 2115355 | 21 |
| 135815256 | 135815263 | 7 | 2117444 | 2117481 | 37 |
| 135817432 | 135817441 | 9 | 2118624 | 2118731 | 107 |
| 135823085 | 135823126 | 41 | 2119404 | 2119563 | 159 |
|  |  |  | 2119709 | 2119774 | 65 |
|  |  |  | 2120690 | 2120694 | 4 |
|  |  |  | 2125247 | 2125297 | 50 |
|  |  |  | 2125443 | 2125459 | 16 |
|  |  |  | 2135116 | 2135120 | 4 |
|  |  |  | 2138042 | 2138185 | 143 |
|  |  |  | 2138227 | 2138236 | 9 |
| **total (14 regions)** | | **741** | **total (21 regions)** | | **1231** |

**S1B: Observed low coverage.**

| ***TSC1*** | | | ***TSC2*** | | |
| --- | --- | --- | --- | --- | --- |
| **start** | **end** | **size (bp)** | **start** | **end** | **size (bp)** |
| 135760461 | 135760757 | 296 | 2088036 | 2088038 | 2 |
| 135764445 | 135764490 | 45 | 2091682 | 2091739 | 57 |
| 135764847 | 135765109 | 262 | 2091673 | 2091922 | 249 |
| 135771169 | 135771196 | 27 | 2092760 | 2093030 | 270 |
| 135771299 | 135771356 | 57 | 2095564 | 2095568 | 4 |
| 135780310 | 135780328 | 18 | 2097643 | 2097936 | 293 |
| 135783803 | 135783821 | 18 | 2099252 | 2099322 | 70 |
| 135783933 | 135783936 | 3 | 2099546 | 2099729 | 183 |
| 135784285 | 135784345 | 60 | 2100661 | 2101140 | 479 |
| 135784382 | 135784422 | 40 | 2101920 | 2101985 | 65 |
| 135786338 | 135786348 | 10 | 2102213 | 2102233 | 20 |
| 135786880 | 135787089 | 209 | 2104965 | 2104990 | 25 |
| 135789092 | 135789368 | 276 | 2105565 | 2105681 | 116 |
| 135789152 | 135789176 | 24 | 2107782 | 2108095 | 313 |
| 135789259 | 135789260 | 1 | 2108095 | 2109810 | 1715 |
| 135790018 | 135790237 | 219 | 2111224 | 2111399 | 175 |
| 135791129 | 135791143 | 14 | 2114839 | 2115334 | 495 |
| 135794807 | 135794838 | 31 | 2115329 | 2115364 | 35 |
| 135797415 | 135797432 | 17 | 2116446 | 2116473 | 27 |
| 135799556 | 135799825 | 269 | 2116869 | 2116897 | 28 |
| 135802111 | 135802262 | 151 | 2117394 | 2117481 | 87 |
| 135802859 | 135803252 | 393 | 2118624 | 2118731 | 107 |
| 135805674 | 135805764 | 90 | 2119558 | 2119710 | 152 |
| 135805937 | 135805984 | 47 | 2119404 | 2119856 | 452 |
| 135808023 | 135808180 | 157 | 2121259 | 2121260 | 1 |
| 135808539 | 135808762 | 223 | 2122735 | 2122764 | 29 |
| 135811592 | 135811632 | 40 | 2123518 | 2123546 | 28 |
| 135812528 | 135812594 | 66 | 2124645 | 2124663 | 18 |
| 135815256 | 135815301 | 45 | 2125069 | 2125100 | 31 |
| 135815556 | 135815567 | 11 | 2125247 | 2125578 | 331 |
| 135817668 | 135817670 | 2 | 2130581 | 2130615 | 34 |
| 135817862 | 135817897 | 35 | 2134380 | 2134393 | 13 |
| 135819692 | 135819744 | 52 | 2137609 | 2137655 | 46 |
| 135820284 | 135820332 | 48 | 2138042 | 2138185 | 143 |
| 135820685 | 135820784 | 99 | 2138631 | 2138686 | 55 |
| 135823085 | 135823159 | 74 |  |  |  |
| **total (36 regions)** | | **3429** | **total (35 regions)** | | **6148** |

**Supporting Table S2. Haloplex custom capture targeted sequencing reads mapped to the *TSC1* and *TSC2* loci using NIMBUS and standard BWA alignment.** Number of reads mapped to the HaloPlex *TSC1* and *TSC2* custom capture regions per individual are shown using both BWA and NIMBUS alignment. Coverage (%) of the *TSC1* and *TSC2* loci at a read-depth > 10 is shown per individual for both the NIMBUS and standard BWA alignment. Expected coverage *TSC1* locus: 98.8%; expected coverage *TSC2* locus: 97.6%.

| **individual** | ***TSC1*** | | | | ***TSC2*** | | | |
| --- | --- | --- | --- | --- | --- | --- | --- | --- |
|  | **NIMBUS** | | **BWA** | | **NIMBUS** | | **BWA** | |
|  | **reads** | **cover-**  **age (%)** | **reads** | **cover-**  **age (%)** | **reads** | **cover-**  **age (%)** | **reads** | **cover-**  **age (%)** |
| **I** | 117054 | 96.4 | 114555 | 88.8 | 125394 | 93.8 | 123405 | 85.2 |
| **II** | 177490 | 97.3 | 174089 | 91.3 | 167228 | 95.0 | 164456 | 92.3 |
| **III** | 173552 | 97.0 | 170526 | 91.0 | 193170 | 93.7 | 190246 | 85.4 |
| **IV** | 104566 | 96.1 | 102509 | 87.5 | 108800 | 92.7 | 107177 | 83.1 |
| **V** | 146408 | 96.5 | 144231 | 89.2 | 127098 | 93.7 | 125045 | 84.3 |
| **VI** | 153320 | 96.8 | 150714 | 90.3 | 161562 | 93.4 | 159416 | 86.9 |
| **VII** | 165840 | 96.9 | 162794 | 91.3 | 160844 | 94.1 | 158247 | 86.2 |

**Supporting Table S3**: **Overview of Haloplex targeted genomic sequencing of the *TSC1* and *TSC2* loci.** The number of heterozygote (het) SNPs and InDels identified at the *TSC1* (S3A) and *TSC2* (S3B) loci are indicated per individual. Possible mosaic changes are indicated in brackets. Cases where an individual was homozygote for the minor allele (hom), and variants detected previously by Sanger sequencing are also shown. “New” variants are those that have not been described previously. ^1.^ screening predominantly with DGGE/SSCP; ^2.^ screening predominantly with Sanger sequencing; ^3.^ Southern blotting; ^4.^ FISH. In all cases MLPA and Q-PCR were performed.

**S3A**: ***TSC1* locus**

| **individual** | **SNP** | | **InDel** | | **GoNL SNP**  **het** | **"new" SNP**  **het** | **"new" InDel**  **het** | **previously identified** |
| --- | --- | --- | --- | --- | --- | --- | --- | --- |
|  | **het** | **hom** | **het** | **hom** |  |  |  |  |
| **I** | 2 | 25 | 2 | 0 | 0 | 4 | 0 | 0 ^1, 3, 4^ |
| **II** | 69 | 13 | 5 | 0 | 2 | 2 (1) | 1 (1) | 3^1^ |
| **III** | 58 | 9 | 2 | 0 | 0 | 0 | 0 | 1^1, 3, 4^ |
| **IV** | 74 | 10 | 7 | 0 | 1 | 0 | 0 | 2^1^ |
| **V** | 1 | 25 | 2 | 0 | 0 | 1 | 1 | 0^2^ |
| **VI** | 0 | 25 | 1 | 1 | 0 | 1 | 0 | 0^2^ |
| **VII** | 32 | 3 | 2 | 0 | 1 | 0 | 0 | 0^2^ |

**S3B: *TSC2* locus**

| **individual** | **SNP** | | **indel** | | **GoNL SNP**  **het** | **"new" SNP**  **het** | **"new" indel**  **het** | **previously identified** |
| --- | --- | --- | --- | --- | --- | --- | --- | --- |
|  | **het** | **hom** | **het** | **hom** |  |  |  |  |
| **I** | 2 | 8 | 0 | 0 | 0 | 4 (1) | 1 (1) | 0^1, 3, 4^ |
| **II** | 11 | 0 | 3 | 0 | 0 | 0 | 1 | 0^1^ |
| **III** | 9 | 1 | 0 | 0 | 2 | 1 (1) | 0 | 0^1, 3, 4^ |
| **IV** | 22 | 19 | 3 | 1 | 0 | 1 | 0 | 2^1^ |
| **V** | 12 | 0 | 3 | 0 | 0 | 0 | 0 | 1^2^ |
| **VI** | 34 | 0 | 2 | 0 | 0 | 1 | 0 | 10^2^ |
| **VII** | 40 (4) | 1 | 5 | 0 | 0 | 4 | 0 | 8^2^ |

**Supporting Table S4. Peak ratios Sanger sequencing.** Relative peak areas for the wild-type and mosaic variant nucleotides were calculated for individuals III and VI and their parents.

| **Individual** | **variant** | **mean peak ratio (mutant/wild-type)** | | | |
| --- | --- | --- | --- | --- | --- |
|  |  | **forward sequence reaction** | **fold change with respect to index** | **reverse sequence reaction** | **fold change with respect to index** |
| **III** | *TSC2* c.3099C>G (p.Y1033*) | 0.057 | 1 | 0.177 | 1 |
| mother of individual **III** | *TSC2* c.3099C>G (p.Y1033*) | 0.004 | 0.07 | 0.094 | 0.53 |
| father of individual **III** | *TSC2* c.3099C>G (p.Y1033*) | 0.005 | 0.09 | 0.073 | 0.41 |
| **VI** | *TSC2* c.2838-122G>A | 0.059 | 1 | 0.205 | 1 |
| mother of individual **VI** | *TSC2* c.2838-122G>A | 0.085 | 1.44 | 0.071 | 0.35 |
| father of individual **VI** | *TSC2* c.2838-122G>A | 0.027 | 0.46 | 0.056 | 0.27 |
